# Factors associated with chronic urticaria in Asian populations: A systematic review and meta-analysis

**DOI:** 10.1016/j.jdin.2026.01.004

**Published:** 2026-01-29

**Authors:** Wanqing Xia, Jia Wang, Xiang Li, Tianci Peng, Bingbing Su, Zhongxing Ouyang, Jiaxuan Tang, Qiyao Xiao, Jiayang Liao, Jialin Deng, Rui Zhou, Cong Peng, Jie Li

**Affiliations:** a Department of Dermatology, Xiangya Hospital, Central South University, Changsha, Hunan, China; b National Clinical Research Center for Geriatric Disorders, Xiangya Hospital, Changsha, Hunan, China; c Hengyang Medical School, University of South China, Hengyang, Hunan, China

**Keywords:** associated conditions, chronic urticaria, helicobacter pylori infection, meta-analysis, risk factors, systematic review, systemic lupus erythematosus

*To the Editor:* Chronic urticaria (CU) is a prevalent dermatologic condition, yet its risk factors in Asian populations remain insufficiently understood.^1^ This study aims to thoroughly assess the potential risk factors influencing the incidence of CU among Asians.

Our study is registered on PROSPERO (CRD420250643142) and reported following Cochrane Handbook and preferred reporting items for systematic reviews and meta-analyses guidelines (Supplementary I, available via Mendeley at https://data.mendeley.com/datasets/hkssw9tmh4/2). We searched PubMed, Embase, and the Cochrane Library for studies published up to October 19, 2024. The search strategy included terms such as “urticaria” or “chronic urticaria” or “angioedema” and “Asia,” along with a series of Asian countries. Studies examining risk factors for CU in Asian populations were included (Fig 1). Due to clinical heterogeneity, we selected the random-effects model to calculate the pooled ORs. Subgroup and sensitivity analyses were conducted to assess heterogeneity and robustness.

**Fig 1:**
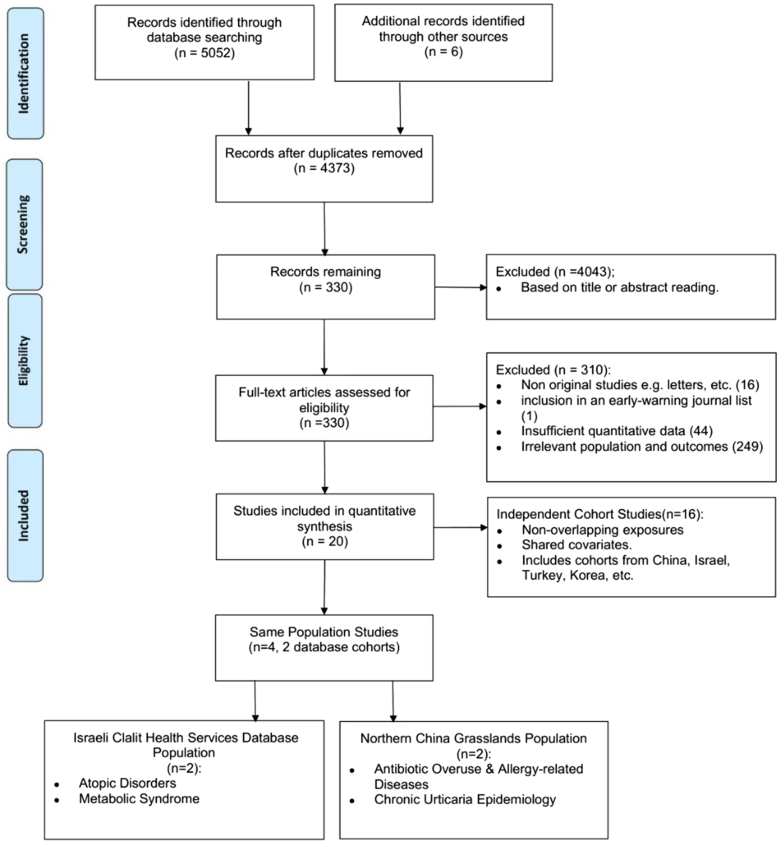
preferred reporting items for systematic reviews and meta-analyses flow diagram of study selection.

Twenty studies involving 6,025,605 participants met the inclusion criteria (Supplementary II, available via Mendeley at https://data.mendeley.com/datasets/hkssw9tmh4/2). We identified 6 patient-related factors: female gender (OR 1.17, 95% confidence interval [CI] 1.01-1.35), allergic rhinitis (OR 2.47, 95% CI 1.19-5.13), systemic lupus erythematosus (OR 3.39, 95% CI 1.62-7.11), rheumatoid arthritis (OR 3.71, 95% CI 1.36-10.11), Helicobacter pylori (HP) infection (OR 2.64, 95% CI 1.59-4.37) and diabetes mellitus (OR 1.79, 95% CI 1.06-3.02), along with one social factor, nonintermediate education level (OR 1.16, 95% CI 1.06-1.27) (Table I). However, neither asthma nor advanced education showed significant associations with CU (Supplementary II, available via Mendeley at https://data.mendeley.com/datasets/hkssw9tmh4/2).

**Table I:** Significant and nonsignificant factors associated with chronic urticaria

Significant factors	No of studies	No of patients	Chronic urticaria OR (95% CI)	Pooled *P* value	I^2^, %	*P* value for Cochran’s Q test	Egger's test *P* value
Gender							
Male			Ref.				
Female	11	5,979,567	1.17 (1.01 to 1.35)	.036	95.3	<.001	.575
Allergic rhinitis							
No			Ref.				
Yes	3	5,786,947	2.47 (1.19 to 5.13)	.015	99.9	<.001	.232
Systemic lupus erythematosus							
No			Ref.				
Yes	3	5,731,952	3.39 (1.62 to 7.11)	.001	93.7	<.001	.896
Rheumatoid arthritis							
No			Ref.				
Yes	3	5,731,952	3.71 (1.36 to 10.11)	.01	98.5	<.001	.955
Hp infection							
No			Ref.				
Yes	5	5,916,290	2.64 (1.59 to 4.37)	<.001	23.7	.263	.914
Diabetes							
No			Ref.				
Yes	5	5,994,735	1.79 (1.06 to 3.02)	.003	98.5	<.001	.446
Non-middle educational level							
Yes			Ref.				
No	3	187,938	1.16 (1.06 to 1.27)	.001	0	.695	.171


Nonsignificant factors	No of studies	No of patients	Chronic urticaria OR (95% CI)	Pooled *P* value	I^2^, %	*P* value for Cochran’s Q test	Egger's test *P* value
Asthma							
No			Ref.				
Yes	3	5,786,947	2.08 (0.84 to 5.13)	.111	99.9	<.001	.034
High educational level							
No			Ref.				
Yes	3	187,938	0.97 (0.86 to 1.09)	.483	86.4	.001	.54

Effect sizes were pooled using the DerSimonian-Laird random-effects model; heterogeneity among studies was assessed via the Q test and I^2^ statistic, with *P* < .05 indicating statistically significant heterogeneity; publication bias was detected using Egger’s test, with *P* < .05 indicating significant publication bias. *95% CI*, 95% Confidence interval; *Hp*, *Helicobacter pylori*; *OR*, odds ratio; *Ref.*, reference group.

A retrospective study showed approximately 17% of CU patients have autoimmune diseases, with female patients developing such diseases within 10 years of urticaria diagnosis.^2^ Thus, we recommend clinical screening of suspected patients for the early detection of severe systemic diseases like systemic lupus erythematosus. Notably, HP infection is also strongly associated with CU^3^, and HP prevalence in the Southeast Asia is higher than the global average.^4^ Despite eradication remains controversial^3^^,^^5^, HP is linked to gastric cancer^4^ and can cause chronic inflammation (eg, gastritis) that impacts CU treatment. We therefore suggest HP screening for patients with digestive symptoms and eradication therapy if positive, consistent with guideline recommendations.^1^

Our study has the following limitations. First, inconsistent outcome reporting, incompatible definitions, and insufficient studies per factor precluded us from conducting subgroup analysis and meta-regression. Notably, the small number of included studies and heterogeneity may affect the robustness of our findings, and subsequent investigations with large-sample sizes and homogeneous methodologies are warranted to validate these results. Second, all included studies are observational in nature, which intrinsically precludes the establishment of a causal relationship between the identified factors and CU. We performed evidence grading and publication bias analysis, with the results presented in Supplementary I, available via Mendeley at https://data.mendeley.com/datasets/hkssw9tmh4/2.

## Conflicts of interest

None disclosed.
